# Supplementary material for: Rapid Detection of SARS-CoV-2 Based on the LAMP Assay Associated with the CRISPRCas12a System
Source: Diagnostics (Basel). 2023 Jun 30;13(13):2233. doi: 10.3390/diagnostics13132233 (PMC10341061; doi:10.3390/diagnostics13132233)
Supplement: Supplementary file 1 [file diagnostics-13-02233-s001.zip › diagnostics-2428273-supplementary.pdf]

Table S1. Primers used for RT-LAMP-only detection

Oligo name	Sequence (5′ – 3′)
F3 SARS-CoV G1- Orf1ab gene	GGAAAGGTTATGGCTGTAGT
FIP SARS-CoV G1- Orf1ab gene	GCAAACCCGTTTAAAAACGATTGTGTGATCAACTCCGCGAAC
FL SARS-CoV G1- Orf1ab gene	GCATCAGCTGACTGAAGCATG
B3 SARS-CoV G1- Orf1ab gene	TCTTGGAAGCGACAACAA
BIP SARS-CoV G1- Orf1ab gene	CGGCACAGGCACTAGTACTGAAAACCAGCTACTTTATCATTG T
BL SARS-CoV G1- Orf1ab gene	GTCGTATACAGGGCTTTTGACA
F3 SARS-CoV G2- Orf1ab gene	CCGCAAGGTTCTTCTTCGTA
FIP SARS-CoV G2- Orf1ab gene	CCAAGCTCGTCGCCTAAGTCAAAAGGAGCTGGTGGCCATAG
FL SARS-CoV G2- Orf1ab gene	TGACTTTAGATCGGCGCCGTAA
B3 SARS-CoV G2- Orf1ab gene	CAGGGCCACAGAAGTTGTT
BIP SARS-CoV G2- Orf1ab gene	AGCAGTGGTGTTACCCGTGAACCGACATAGCGAGTGTATGCC
BL SARS-CoV G2- Orf1ab gene	ATGCGTGAGCTTAACGGAGG
F3 SARS-CoV G3- N gene	TCGCAATGGCTTGTCTTGT
FIP SARS-CoV G3- N gene	ACCACATGGAACGCGTACGCGCTTGATGTGGCTCAGCT
FL SARS-CoV G3- N gene	GCAAACAGTCTGAAAGAAGCAATGA
B3 SARS-CoV G3- N gene	TCTTGGAAGCGACAACAA
BIP SARS-CoV G3- N gene	CGGCACAGGCACTAGTACTGAAAACCAGCTACTTTATCATTG T
BL SARS-CoV G3- N gene	GTCGTATACAGGGCTTTTGACA
F3 RNase P POP7	TTGATGAGCTGGAGCCA
B3 RNase P POP7	CACCCTCAATGCAGAGTC
FIP RNase P POP7	GTGTGACCCTGAAGACTCGGTTTTAGCCACTGACTCGGATC
BIP RNase P POP7	CCTCCGTGATATGGCTCTTCGTTTTTTTCTTACATGGCTCTGGTC
LF RNase P POP7	ATGTGGATGGCTGAGTTGTT
LB RNase P POP7	CATGCTGAGTACTGGACCTC

Table S2. gRNA and reporters used for CRISPR-Cas12a-based SARS-CoV-2 detection.

Orf1ab-gene gRNA	UAAUUUCUACUAAGUGUAGUAAAGAAAACUGGAGGAACACUAAAC	gRNA for the CRISPR-Cas12a system
RNaseP POP7 gRNA	UAAUUUCUACUAAGUGUAGAUAAUUACUUGGGUGUGACCCU	
6-FAMBHQ	/56-FAM/TTTTTTTTTTTT/3BHQ_1/	For fluorescence assay
6-FAMBIOTIN	/56-FAM/TTATTATT/3Bio/	For lateral flow assay
